# Global, regional, and national burden of ovarian cancer due to high BMI, 1990–2021 and projections to 2050: a systematic analysis based on the global burden of disease 2021 study

**DOI:** 10.3389/fnut.2026.1688767

**Published:** 2026-02-09

**Authors:** Shuiqing Xu, Xiaotong Fu, Ming Wang

**Affiliations:** 1 Department of Gynecologic Oncology, Beijing Obstetrics and Gynecology Hospital, Capital Medical University/Beijing Maternal and Child Health Care Hospital, Beijing, China; 2 Department of Neurology, Beijing Chaoyang Hospital of Captital Medical University, Beijing, China

**Keywords:** disease burden, global trends, high BMI, ovarian cancer, projections

## Abstract

Using GBD 2021 data, this study quantifies the disease burden of ovarian cancer attributable to high BMI—encompassing mortality and disability-adjusted life years (DALYs)—across 204 countries/regions globally between 1990 and 2021. By analyzing temporal trends, we will identify regions with a significant increase in burden, providing a basis for formulating targeted prevention and control strategies. We also explore correlations between disease burden and socioeconomic indicators to assess how socioeconomic factors influence ovarian cancer incidence and prognosis. In addition, we will analyze the roles of aging, population structure changes, and epidemiological factors in the burden of ovarian cancer caused by high BMI, dissect differences between countries, and predict trends up to 2050. The results of this study will provide important references for public health policy formulation, rational resource allocation, and the prevention and control of ovarian cancer.

## 1 Introduction

Ovarian cancer (OC) is one of the most common gynecological malignancies worldwide, ranking among the top three in incidence in many regions, and is recognized as the most deadly gynecological cancer with the highest global mortality rate (1). This grim outcome stems from its insidious onset, lack of specific early symptoms, and absence of highly sensitive screening biomarkers—factors that lead approximately 70% of patients to be diagnosed at an advanced stage, resulting in poor overall prognosis (1–3). Currently, the standard first-line treatment for advanced ovarian cancer involves cytoreductive surgery combined with platinum-based chemotherapy and targeted maintenance therapy, however, the 5-year survival rate remains only 51.6% (4).

Advancing age is the most prominent risk factor for ovarian cancer: the incidence rate rises from 15.7 to 54 per 100,000 individuals between the ages of 40 and 79, with a mean age at diagnosis of 59 years. Familial history represents the strongest non-age-related risk factor for the disease (5). Notably, ovarian cancer incidence exhibits substantial geographic disparities, with notably higher rates in regions such as Northern Europe and North America, which is closely linked to the interplay of genetic susceptibility, environmental exposures, and lifestyle factors (1). The elevated incidence in Northern Europe and North America is primarily driven by four key factors: first, genetic predisposition, as these populations have a significantly higher carriage rate of BRCA1/2 germline mutations compared to other regions (6); second, the prevalence of obesity—with adult obesity rates reaching 42% in North America and 28% in Northern Europe—high BMI increases ovarian cancer risk by 12–24% through mechanisms including hormonal dysregulation and chronic inflammation, and the proportion of high BMI-related ovarian cancer cases (18.3%) in these regions is substantially higher than the global average (7); third, reproduction-related factors such as advanced age at first childbirth and high childlessness rates (5, 8); fourth, well-established screening systems and cancer registries that enhance the detection of early asymptomatic cases, reducing underdiagnosis and ensuring more accurate incidence statistic (3).

The geographic variations in ovarian cancer incidence—such as the notably higher rates observed in regions like Northern Europe and North America—underscore the multifactorial nature of the disease burden. Among these contributing factors, high BMI stands out as a key modifiable risk factor that plays a critical role in driving ovarian cancer burden globally, particularly in these high-incidence regions (3, 5). It is important to clarify that while ‘high BMI’ and ‘obesity’ are often referenced in epidemiological research, they represent a continuum of adiposity rather than interchangeable terms: high BMI is a quantitative measure (typically defined as BMI ≥ 25 kg/m^2^ per the World Health Organization [WHO] classification, encompassing both overweight [25–29.9 kg/m^2^] and obesity [≥30 kg/m^2^]), whereas obesity specifically denotes excessive adipose tissue accumulation at the upper end of this spectrum (BMI ≥ 30 kg/m^2^) (9). This distinction aligns with our focus, as both overweight and obesity (i.e., the broader high BMI category) have been consistently linked to elevated ovarian cancer risk, with dose-dependent effects across BMI strata (10). This highlights the value of targeting high BMI in its entirety—not just obesity—in ovarian cancer prevention strategies to address the growing disease burden. A large body of research has established that obesity (as a subset of high BMI) is closely associated with cancer occurrence and progression (4, 11) with underlying mechanisms including chronic inflammation induction, hormonal microenvironment alterations (e.g., elevated estrogen and insulin levels), and subsequent promotion of cancer cell proliferation and invasion (12–14). Additionally, obesity impairs immune function, reducing the body’s capacity for tumor cell surveillance and elimination (15) and these mechanistic pathways are similarly relevant to overweight individuals (BMI 25–29.9 kg/m^2^), albeit potentially to a lesser degree. Collectively, this evidence confirms high BMI as a critical contributor to ovarian cancer development and progression, translating to a substantial global burden of ovarian cancer attributable to high BMI (encompassing both overweight and obesity). While the Global Burden of Disease (GBD) study offers comprehensive data support for disease burden assessment, most prior research on ovarian cancer has centered on overall burden, with relatively limited attention to the specific burden linked to high BMI. As such, further clarification is needed regarding its distribution across countries/regions, temporal trends, and associations with socioeconomic factors.

Using GBD 2021 data, this study quantifies the disease burden of ovarian cancer attributable to high BMI—encompassing mortality and disability-adjusted life years (DALYs)—across 204 countries/regions globally between 1990 and 2021. By analyzing temporal trends, we will identify regions with a significant increase in burden, providing a basis for formulating targeted prevention and control strategies. We also explore correlations between disease burden and socioeconomic indicators to assess how socioeconomic factors influence ovarian cancer incidence and prognosis. In addition, we will analyze the roles of aging, population structure changes, and epidemiological factors in the burden of ovarian cancer caused by high BMI, dissect differences between countries, and predict trends up to 2050. The results of this study will provide important references for public health policy formulation, rational resource allocation, and the prevention and control of ovarian cancer.

## 2 Methods

### 2.1 Data sources

To address the global variation and long-term trends of high BMI-attributable ovarian cancer burden, data were extracted from the GBD 2021 database—an authoritative source integrating disease burden and injury data from 204 countries/regions (1990–2021) that explicitly classifies BMI as an attributable risk factor for ovarian cancer. Data extraction was conducted via the official GBD visualization platform, with geographical stratification as follows: global scope, five Social Demographic Index (SDI) tiers (low, low-middle, middle, high-middle, high), 21 GBD-specific regions, and 204 individual countries/regions. Core metrics included mortality, disability-adjusted life years (DALYs), and their 95% uncertainty intervals (UIs) for high BMI-attributable ovarian cancer, used to systematically quantify disease burden.

### 2.2 Statistical analysis

#### 2.2.1 Burden description

We quantified the burden of ovarian cancer attributable to high BMI using four core epidemiological metrics, consistent with the Global Burden of Disease (GBD) 2021 standard analytical framework. These metrics are defined as follows: (1) Mortality: The number of deaths directly attributed to high BMI-related ovarian cancer in a given population and time period; (2) Disability-Adjusted Life Years (DALYs): A composite metric integrating years of life lost (YLLs) due to premature death and years lived with disability (YLDs) from the disease, quantifying the overall health loss caused by high BMI-attributable ovarian cancer; (3) Age-Standardized Mortality Rate (ASMR): Mortality adjusted by the World Health Organization (WHO) standard population weights to eliminate the confounding effect of cross-regional differences in population age structure, calculated as (age-specific mortality/corresponding population size) × standard population weights; and (4) Age-Standardized DALY Rate (ASDR): DALYs standardized using the same WHO standard population weights, enabling comparable assessment of health loss burden across regions with distinct demographic structures.

#### 2.2.2 Trend analysis

Age-standardized disease burden rates were computed across ages, regions, and countries. Temporal trends were quantified using estimated annual percentage change (EAPC) and further refined via the Joinpoint regression model (version 4.9.0.0)—a tool widely used for identifying inflection points in time-series data—to explore trends in high BMI-attributable ovarian cancer burden (1990–2021). The model generated average annual percentage changes (AAPC) and segment-specific annual percentage changes (APC), with trend direction assessed by the 95% confidence interval (CI) relative to zero: upward (entire CI > 0), downward (entire CI < 0), or stable (CI spans zero). Model fitting and AAPC/APC calculation were performed in Joinpoint 4.9.0.0, with results visualized in R.

#### 2.2.3 Correlation analysis

To explore how age and socioeconomic development influence high BMI-attributable ovarian cancer burden (consistent with study background), two correlation analyses were conducted: (1) Age-related correlation: Based on GBD data, dual-axis age plots (mortality vs. death counts) were used to visualize age-specific mortality and DALYs, with 95% CIs reflecting uncertainty; Spearman rank correlation coefficient quantified the independent association between age and these metrics. (2) SDI-related correlation: Across 21 regions and 204 countries, scatter plots and correlation coefficients examined relationships between SDI and EAPC of ASMR/ASDR, with significance testing performed.

#### 2.2.4 Prediction analysis

We used the Bayesian Age-Period-Cohort (BAPC) model to project the ovarian cancer burden attributable to high BMI for different age groups in 2050 under a no-intervention scenario. This model extends the traditional APC model by incorporating Bayesian Markov chain Monte Carlo (MCMC) algorithms, enabling flexible modeling of complex temporal trends and improved handling of missing data and parameter uncertainty. The key assumptions of the BAPC model are: (1) Stationarity of age, period, and cohort effects; (2) Mutual independence of age, period, and cohort effects; (3) Linear relationship between high BMI and ovarian cancer risk; (4) Use of Bayesian priors; and (5) Projection under a no-intervention scenario.

#### 2.2.5 Decomposition analysis

To project future high BMI-attributable ovarian cancer burden (2050) for different age groups, we used the Bayesian Age-Period-Cohort (BAPC) model (Keyfitz, 1986)—a widely cited extension of the traditional APC model that integrates Bayesian Markov chain Monte Carlo (MCMC) algorithms, facilitating flexible modeling of complex temporal trends and robust handling of missing data/parameter uncertainty. Key model assumptions: (1) Stationarity of age, period, and cohort effects; (2) Mutual independence of these effects; (3) Linear high BMI-ovarian cancer risk relationship; (4) Specification of Bayesian priors (based on existing epidemiological evidence); (5) Projection under a no-intervention scenario (baseline projection for intervention comparison).

#### 2.2.6 Health inequality analysis

To assess health inequalities in high BMI-attributable ovarian cancer burden across socioeconomic strata, we analyzed cause-of-death, demographic, and SDI data (1990, 2021) from the GBD database. Populations were ranked by SDI, with cumulative proportions of population, mortality, and DALYs calculated to generate Lorenz-like curves. The concentration index (SII) quantified and visualized burden disparities across SDI strata. Data were bias-adjusted using the DisMod-MR model, and nonparametric methods were employed to calculate 95% CIs, quantifying uncertainty.

#### 2.2.7 Frontier analysis

This study employed frontier analysis to systematically compare the performance of countries in managing the ovarian cancer burden attributable to high BMI (The ‘frontier’ herein refers to a stochastic boundary derived from parametric stochastic frontier analysis (SFA), integrating a regression-based theoretical minimum DALY rate for a given SDI and random variability, rather than the lowest observed DALY rate). We introduced a key metric, the ‘effective gap’, which denotes the discrepancy between observed burden and the model-predicted potential burden based on SDI levels. This metric reflects the gap between a country’s current performance and the ideal state of high BMI-related ovarian cancer burden control, while identifying top-performing countries/regions. This benchmark establishes an optimal reference system, providing clear targets and models for other countries/regions to follow.

## 3 Results

### 3.1 Global burden of ovarian cancer attributable to high BMI

At the global level, the number of death cases increased from 6,850 (95% UI: 1,423–12,865) in 1990 to 17,344 (95% UI: 4,141–30,810) in 2021 (Table 1), while the number of DALY cases increased from 188,874 (95% UI: 28,401–355,691) to 477,248 (95% UI: 113,449–840,002) in 2021 (Table 2). ASMR and ASDR increased from 0.32 (95% UI: 0.07–0.61) and 8.72 (95% UI: 1.78–16.41) per 100,000 in 1990 to 0.38 (95% UI: 0.09–0.67) and 10.56 (95% UI, 2.50–18.57) per 100,000 in 2021, respectively. Temporal trend analysis revealed positive values for the Estimated Annual Percentage Change (EAPC) associated with ASMR and ASDR due to high BMI-related ovarian cancer, at 0.40 (95% CI, 0.32–0.47) and 0.51 (95% CI, 0.45–0.57), respectively. This suggests a general upward trend in the global burden of ovarian cancer due to high BMI (Tables 1, 2).

**Table 1:** Deaths and ASMR of ovarian cancer attributable to the high BMI in 1990 and 2021 and the EAPC from 1990 to 2021.

Deaths	1990		2021		EAPC (1990–2021)
	Death cases	ASMR (per 100,000)	Death cases	ASMR (per 100,000)	ASMR (per 100,000)
Location	No. (95%UI)	No. (95%UI)	No. (95%UI)	No. (95%UI)	No. (95%CI)
Global	6,850.05 (1,422.80, 12,864.70)	0.32 (0.07, 0.61)	17,344.47 (4,141.33, 30,810.14)	0.38 (0.09, 0.67)	0.40 (0.32, 0.47)
High SDI	3,801.70 (811.75, 7,120.06)	0.61 (0.13, 1.15)	6,186.80 (1,531.13, 10,979.34)	0.57 (0.14, 1.01)	−0.36 (−0.50, −0.22)
High-middle SDI	2,243.75 (483.65, 4,206.94)	0.40 (0.09, 0.75)	5,094.95 (1,240.98, 9,059.80)	0.48 (0.12, 0.85)	0.47 (0.37, 0.58)
Middle SDI	542.54 (65.34, 1,064.41)	0.10 (0.01, 0.19)	3,832.58 (903.66, 6,971.62)	0.26 (0.06, 0.48)	3.16 (3.08, 3.24)
Low-middle SDI	187.03 (13.39, 391.74)	0.06 (0.00, 0.12)	1,771.25 (357.16, 3,219.98)	0.22 (0.04, 0.41)	4.69 (4.48, 4.90)
Low SDI	62.43 (0.00, 138.85)	0.05 (−0.00, 0.11)	432.55 (67.53, 839.64)	0.15 (0.02, 0.29)	3.61 (3.51, 3.70)
High-income Asia Pacific	84.58 (−8.64, 188.99)	0.07 (−0.01, 0.17)	252.87 (21.85, 508.19)	0.12 (0.01, 0.24)	1.27 (1.14, 1.40)
High-income North America	1,604.42 (382.12, 2,944.24)	0.83 (0.20, 1.52)	2,701.48 (726.13, 4,684.52)	0.78 (0.21, 1.34)	−0.35 (−0.60, −0.10)
East Asia	144.64 (−70.01, 391.94)	0.03 (−0.02, 0.08)	1,814.16 (355.59, 3,762.83)	0.16 (0.03, 0.33)	5.04 (4.87, 5.21)
Southeast Asia	72.77 (−4.69, 160.49)	0.05 (−0.00, 0.10)	762.87 (142.29, 1,448.82)	0.20 (0.04, 0.38)	4.96 (4.68, 5.25)
Oceania	1.73 (0.33, 3.53)	0.11 (0.02, 0.22)	7.85 (2.05, 14.59)	0.19 (0.05, 0.34)	1.91 (1.76, 2.06)
Central Asia	81.68 (17.92, 150.90)	0.29 (0.06, 0.54)	254.80 (62.21, 458.90)	0.53 (0.13, 0.95)	1.95 (1.79, 2.11)
Central Europe	594.79 (137.60, 1,094.62)	0.70 (0.16, 1.30)	1,137.68 (294.75, 2,060.05)	0.94 (0.24, 1.71)	0.95 (0.76, 1.14)
Eastern Europe	1,181.87 (280.29, 2,096.22)	0.68 (0.16, 1.21)	1,832.12 (491.01, 3,169.15)	0.89 (0.24, 1.54)	0.81 (0.61, 1.01)
Australasia	106.75 (22.28, 197.62)	0.85 (0.18, 1.58)	172.94 (44.53, 314.78)	0.62 (0.16, 1.10)	−0.96 (−1.36, −0.57)
Western Europe	2,051.28 (420.81, 3,876.48)	0.63 (0.13, 1.20)	2,895.31 (670.73, 5,328.72)	0.58 (0.14, 1.06)	−0.39 (−0.49, −0.30)
Southern Latin America	151.62 (35.38, 285.87)	0.59 (0.14, 1.12)	319.58 (83.97, 567.00)	0.68 (0.18, 1.20)	0.65 (0.47, 0.84)
Caribbean	29.96 (6.29, 56.78)	0.22 (0.05, 0.42)	130.41 (31.24, 238.34)	0.46 (0.11, 0.84)	2.32 (2.10, 2.54)
Andean Latin America	18.30 (3.04, 37.75)	0.16 (0.03, 0.33)	158.26 (39.30, 305.29)	0.50 (0.12, 0.97)	3.76 (3.33, 4.20)
Central Latin America	144.51 (31.40, 271.17)	0.32 (0.07, 0.59)	923.57 (262.13, 1,669.17)	0.67 (0.19, 1.21)	2.40 (2.30, 2.50)
Tropical Latin America	145.13 (29.92, 282.05)	0.28 (0.06, 0.55)	645.69 (150.26, 1,192.46)	0.46 (0.11, 0.84)	1.24 (1.10, 1.37)
North Africa and Middle East	215.03 (43.99, 427.39)	0.24 (0.05, 0.49)	1,200.46 (345.20, 2,095.69)	0.51 (0.15, 0.89)	2.53 (2.46, 2.60)
South Asia	92.75 (−16.74, 213.54)	0.03 (−0.01, 0.07)	1,302.78 (208.22, 2,414.01)	0.16 (0.03, 0.30)	5.86 (5.70, 6.02)
Central Sub-Saharan Africa	6.08 (0.11, 13.85)	0.05 (0.00, 0.10)	61.32 (10.11, 125.02)	0.18 (0.03, 0.37)	4.70 (4.52, 4.87)
Eastern Sub-Saharan Africa	33.02 (−0.10, 70.34)	0.08 (−0.00, 0.17)	252.78 (41.93, 497.61)	0.25 (0.04, 0.49)	3.84 (3.80, 3.89)
Southern Sub-Saharan Africa	56.20 (14.07, 106.79)	0.36 (0.09, 0.68)	278.23 (76.36, 493.63)	0.81 (0.22, 1.43)	2.89 (2.77, 3.01)
Western Sub-Saharan Africa	32.93 (5.00, 61.48)	0.07 (0.01, 0.14)	239.31 (50.93, 462.37)	0.21 (0.05, 0.41)	3.44 (3.37, 3.50)

No., number; ASMR, age-standardized mortality rate; UI, uncertainty interval; EAPC, estimated annual percentage change; CI, confidential interval; SDI, socio-demographic index.

**Table 2:** DALYs and ASDR of ovarian cancer attributable to the high BMI in 1990 and 2021 and the EAPC from 1990 to 2021.

Deaths	1990		2021		EAPC (1990–2021)
	DALYs cases	ASDR (per 100,000)	DALYs cases	ASDR (per 100,000)	ASDR
Location	No. (95%UI)	No. (95%UI)	No. (95%UI)	No. (95%UI)	No. (95%CI)
Global	188,874.09 (38,400.57, 355,691.42)	8.72 (1.78, 16.41)	477,248.38 (113,449.26, 840,002.14)	10.56 (2.50, 18.57)	0.51 (0.45, 0.57)
High SDI	96,741.78 (20,587.01, 180,827.63)	16.78 (3.57, 31.36)	144,449.46 (360,80.39, 255,582.85)	15.13 (3.79, 26.82)	−0.43 (−0.56, −0.30)
High-middle SDI	65,388.64 (13,955.94, 121,986.45)	11.71 (2.49, 21.84)	138,126.25 (33,461.41, 244,870.67)	13.54 (3.26, 24.13)	0.33 (0.25, 0.42)
Middle SDI	17,976.12 (2,083.04, 35,888.37)	2.99 (0.36, 5.90)	121,138.56 (28,726.80, 221,614.16)	8.25 (1.95, 15.09)	3.20 (3.13, 3.26)
Low-middle SDI	6,283.22 (459.39, 13,116.94)	1.78 (0.13, 3.74)	57,905.91 (11,972.87, 104,670.77)	6.96 (1.43, 12.58)	4.69 (4.48, 4.90)
Low SDI	2,127.12 (24.10, 4,780.70)	1.60 (0.01, 3.56)	14,942.61 (2,348.90, 28,748.14)	4.68 (0.74, 9.04)	3.55 (3.45, 3.65)
High-income Asia Pacific	2,550.45 (−282.64, 5,764.78)	2.29 (−0.28, 5.19)	6,116.91 (662.27, 12,206.52)	3.50 (0.38, 6.99)	1.21 (1.08, 1.34)
High-income North America	41,346.22 (10,085.33, 74,963.22)	23.36 (5.74, 42.27)	64,190.40 (17,683.61, 111,223.47)	20.45 (5.66, 35.38)	−0.54 (−0.76, −0.32)
East Asia	4,730.85 (−2,392.11, 12,911.04)	0.97 (−0.47, 2.63)	54,989.10 (10,888.91, 112,924.07)	4.86 (0.96, 9.99)	4.99 (4.84, 5.14)
Southeast Asia	2,758.92 (−82.96, 6,007.07)	1.67 (−0.06, 3.64)	26,640.24 (5,006.87, 50,775.37)	6.82 (1.27, 12.97)	4.74 (4.44, 5.04)
Oceania	62.43 (12.36, 126.02)	3.41 (0.66, 6.95)	282.52 (73.05, 533.89)	5.93 (1.54, 11.06)	1.84 (1.68, 2.00)
Central Asia	2,522.32 (553.92, 4,677.23)	9.08 (2.00, 16.79)	7,936.34 (1,896.10, 14,299.95)	15.77 (3.76, 28.49)	1.81 (1.64, 1.98)
Central Europe	16,872.60 (38,40.92, 31,014.49)	20.59 (4.65, 38.13)	27,712.57 (7,172.31, 50,229.68)	25.65 (6.53, 46.23)	0.72 (0.52, 0.92)
Eastern Europe	35,232.83 (8,317.94, 62,486.22)	21.36 (4.99, 37.80)	50,055.73 (13,203.03, 86,237.98)	26.48 (6.87, 45.59)	0.59 (0.39, 0.78)
Australasia	2,815.67 (599.25, 5,213.53)	23.68 (5.00, 43.97)	3,935.33 (1,040.69, 7,040.62)	15.67 (4.12, 27.86)	−1.25 (−1.64, −0.87)
Western Europe	50,346.11 (10,327.68, 95,161.15)	17.18 (3.53, 32.53)	62,381.03 (14,564.22, 114,043.85)	14.70 (3.42, 26.90)	−0.62 (−0.73, −0.52)
Southern Latin America	4,240.53 (978.24, 7,969.77)	16.81 (3.87, 31.61)	8,626.68 (2,304.97, 15,146.62)	19.48 (5.21, 34.22)	0.67 (0.49, 0.84)
Caribbean	951.47 (199.37, 1,788.06)	6.81 (1.44, 12.82)	3,937.04 (949.55, 7,222.06)	14.23 (3.43, 26.10)	2.28 (2.06, 2.51)
Andean Latin America	620.15 (111.23, 1,285.19)	5.20 (0.93, 10.80)	5,018.35 (1,244.55, 9,643.33)	15.55 (3.86, 29.85)	3.68 (3.24, 4.12)
Central Latin America	4,750.06 (1,032.77, 8,901.10)	9.51 (2.09, 17.81)	29,277.40 (8,417.92, 52,488.18)	20.87 (5.99, 37.44)	2.52 (2.43, 2.61)
Tropical Latin America	4,626.21 (971.66, 9,022.50)	8.52 (1.78, 16.56)	19,031.60 (4,476.31, 34,718.97)	13.52 (3.18, 24.66)	1.18 (1.06, 1.29)
North Africa and Middle East	6,993.91 (1,427.74, 13,918.82)	7.27 (1.49, 14.33)	37,560.42 (10,820.44, 65,762.34)	14.62 (4.22, 25.60)	2.37 (2.31, 2.42)
South Asia	3,319.66 (−476.81, 7,496.99)	0.98 (−0.16, 2.23)	42,615.10 (68,15.35, 78,757.43)	5.11 (0.82, 9.43)	5.61 (5.48, 5.75)
Central Sub-Saharan Africa	194.89 (0.91, 451.03)	1.35 (0.01, 3.08)	2,048.37 (345.33, 4,184.32)	5.55 (0.92, 11.34)	4.73 (4.56, 4.91)
Eastern Sub-Saharan Africa	1,133.35 (11.78, 2,412.22)	2.48 (0.02, 5.26)	8,753.46 (1,482.77, 17,322.79)	7.84 (1.32, 15.40)	3.74 (3.69, 3.79)
Southern Sub-Saharan Africa	1,774.40 (442.33, 3,342.51)	10.58 (2.68, 19.98)	8,403.83 (2,318.31, 14,968.63)	23.20 (6.41, 41.25)	2.79 (2.67, 2.90)
Western Sub-Saharan Africa	1,031.05 (162.33, 1,904.76)	2.18 (0.34, 4.04)	7,735.96 (1,632.74, 14,871.56)	6.12 (1.30, 11.80)	3.34 (3.28, 3.41)

No., number; DALYs, disability adjusted life years; ASDR, age-standardized DALY rate; UI, uncertainty interval; EAPC, estimated annual percentage change; CI, confidential interval; SDI, socio-demographic index.

### 3.2 Regional burden of ovarian cancer attributable to high BMI

At the regional level, in 2021, Western Europe had the highest number of ovarian cancer deaths attributed to high BMI, with 2,895 deaths (95% UI: 671–5,329), while the region with the highest number of DALY cases was High-income North America, with 64,190 DALYs (95% UI: 17,684–111,223). In contrast, Oceania had the lowest number of deaths (*n* = 8; 95% UI: 2–15) and DALYs (*n* = 283; 95% UI: 73–534). However, However, the highest ASMR (0.94 per 100,000; 95% UI: 0.24–1.71) and ASDR (7.84 per 100,000; 95% UI: 1.32–15.40) were observed in Central Europe and Eastern Sub-Saharan Africa, respectively. At the same time, the lowest ASMR was observed in High-income Asia Pacific, with a rate of 0.12 per 100,000 people (95% UI: 0.01–0.24), and the lowest ASDR was found in Tropical Latin America, with a rate of 13.52 per 100,000 people (95% UI: 3.18–24.66). From 1990 to 2021, the regional gap in ASMR and ASDR widened significantly. South Asia had the largest increase (ASMR-EAPC = 5.86, 95% CI: 5.70–6.02; ASDR-EAPC = 5.61, 95% CI: 5.48–5.75), while Australasia showed the largest decrease (ASMR-EAPC = −0.96, 95% CI: −1.36 to −0.57; ASDR-EAPC = −1.25, 95% CI: −1.64 to −0.87) (Tables 1, 2).

**Figure 1:**
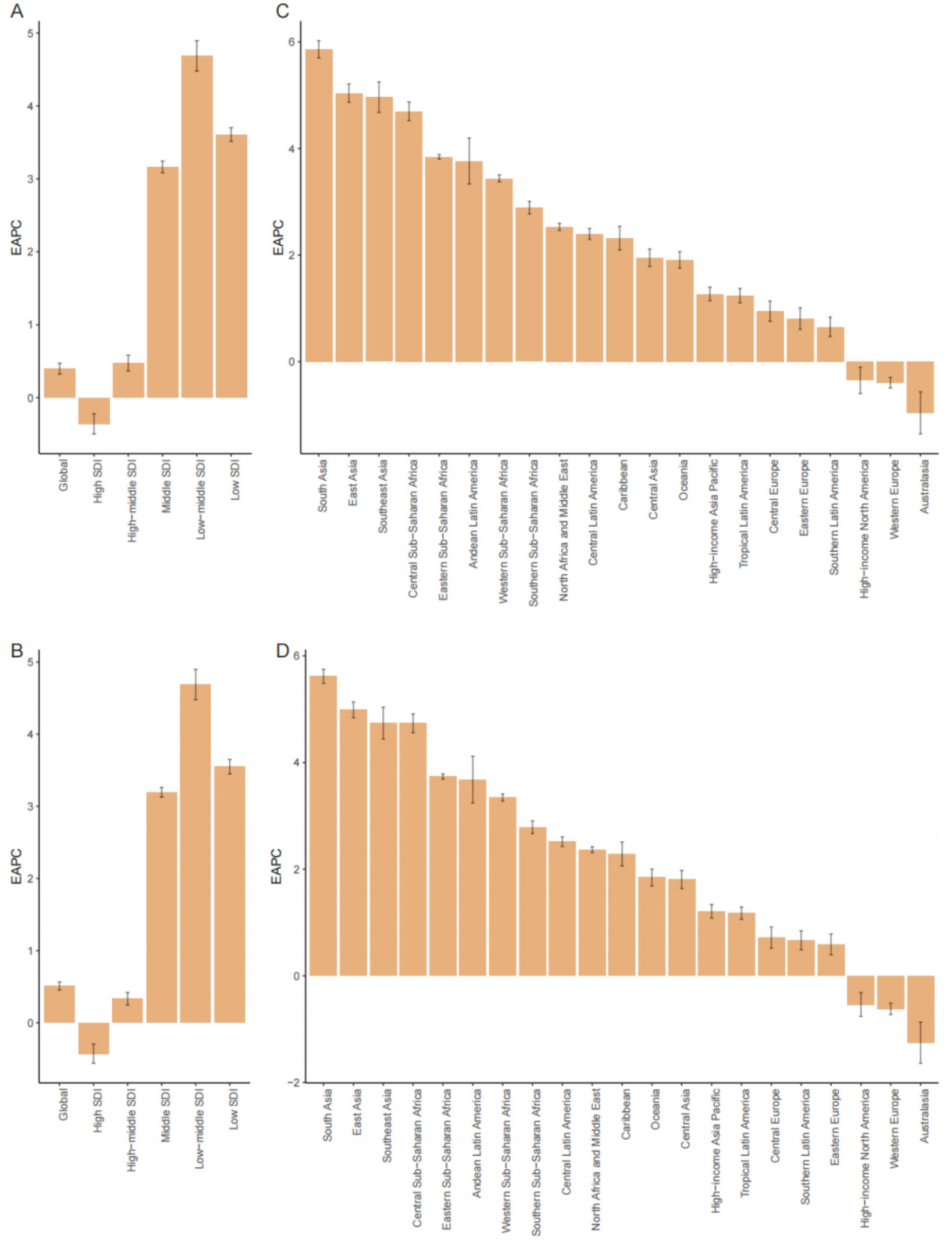
Distribution of EAPC in ASMR and ASDR for ovarian cancer attributable to high BMI across 5 SDI regions and 21 GBD regions, 1990–2021 **(A–D)**. **(A)** EAPC in ASMR across 5 SDI regions; **(B)** APC in ASDR across 5 SDI regions; **(C)** EAPC in ASMR across 21 GBD regions; **(D)** EAPC in ASDR across 21 GBD regions.

In the five SDI regions, high SDI was associated with the highest number of ovarian cancer deaths and DALY cases attributable to high BMI in 2021 (144,449 cases, 95% UI: 36,080–255,583), while low SDI regions had the lowest (433 deaths, 95% UI: 68–840; 5 DALY cases, 95% UI: 1–9), respectively (Tables 1, 2). From 1990 to 2021, except for a significant decrease in ASMR and ASDR in high SDI regions, ASMR and ASDR significantly increased in other SDI regions, with the most notable increase observed in the low-middle SDI regions (Tables 1, 2; Figure 1.)

### 3.3 National burden of ovarian cancer attributable to high BMI

At the national level, in 2021, the top five countries with the highest number of deaths and DALY cases related to ovarian cancer attributable to high BMI were the United States of America, China, the Russian Federation, India, and Brazil. The death counts for these countries were 2,464 (95% UI: 666–4,274), 1,744 (95% UI: 342–3,601), 1,330 (95% UI: 362–2,327), 910 (95% UI: 153–1,740), and 634 (95% UI: 148–1,170), respectively. The corresponding DALY cases were 58,749 (95% UI: 16,253–101,446), 52,980 (95% UI: 10,497–108,333), 36,074 (95% UI: 9,766–62,744), 28,797 (95% UI: 4,800–55,470), and 18,672 (95% UI: 4,399–34,138), respectively. Additionally, in 2021, the United Arab Emirates had the highest ASMR and ASDR due to high BMI-related ovarian cancer, with rates of 3.73 (95% UI: 1.05–6.55) and 80.08 (95% UI: 22.77–140.04), respectively.

**Figure 2:**
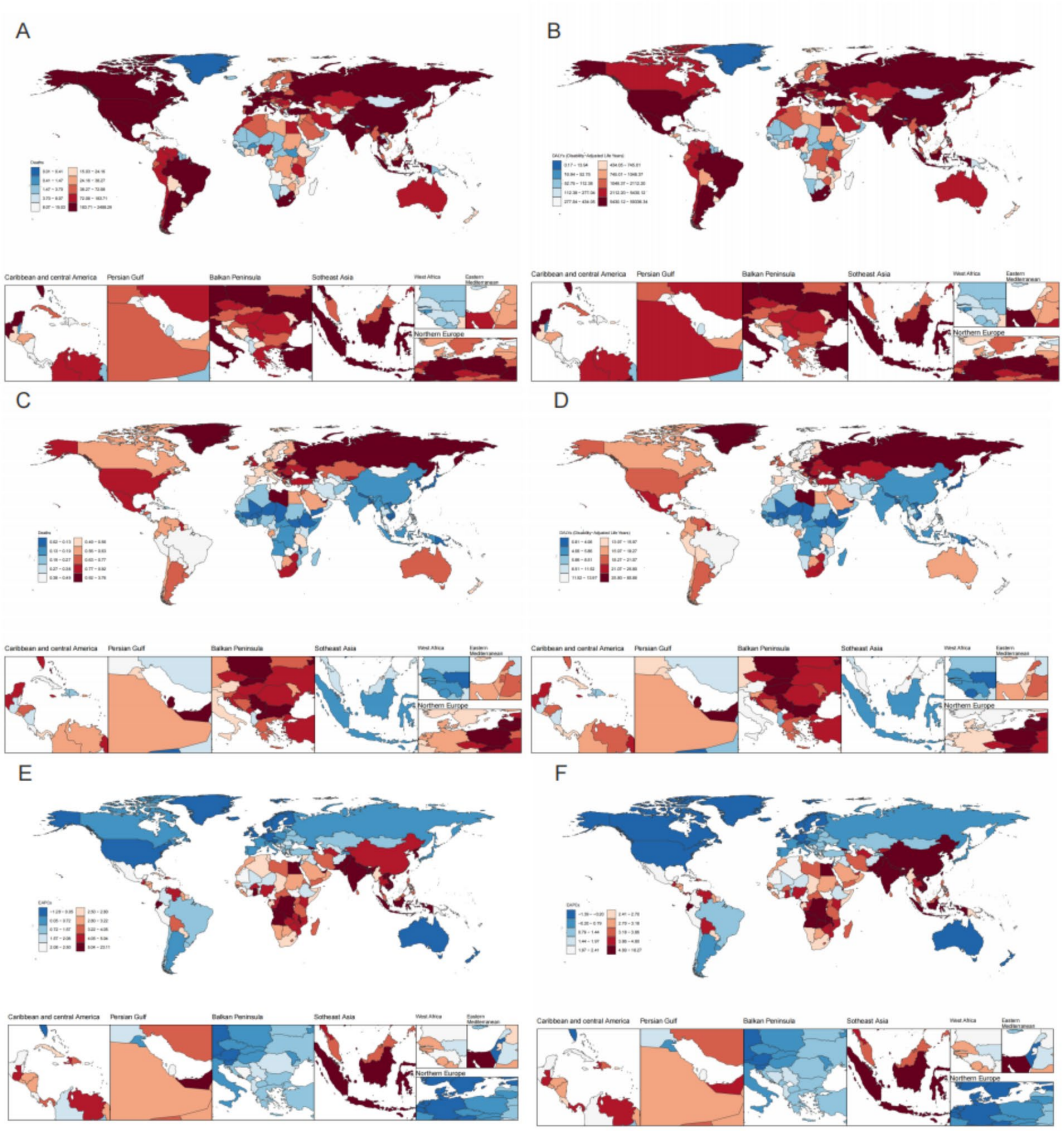
Maps of death numbers and DALYs by country/region in 2021. **(A, C, E)** Death numbers, ASMR, and EAPC in ASMR by country/region in 2021; **(B, D, F)** DALYs, ASDR, and EAPC in ASDR by country/region in 2021.

Time trend analysis showed that Timor-Leste had the most substantial increases in ASMR (EAPC = 15.46; 95% CI: 14.50–16.43) and ASDR (EAPC = 18.09; 95% CI: 15.96–20.27), followed by Vietnam and Bangladesh. Greenland experienced the largest decrease in ASMR due to high BMI-related ovarian cancer, with an EAPC of −1.27 (95% CI: −1.55 to −0.99), followed by Germany and New Zealand. However, the largest decrease in ASDR was observed in Sweden, with an EAPC of −1.38 (95% CI: −1.64 to −1.11), followed by New Zealand and Germany (Figure 2; Supplementary Tables 1, 2).

### 3.4 Age-period trends in ovarian cancer burden attributable to high BMI

**Figure 3:**
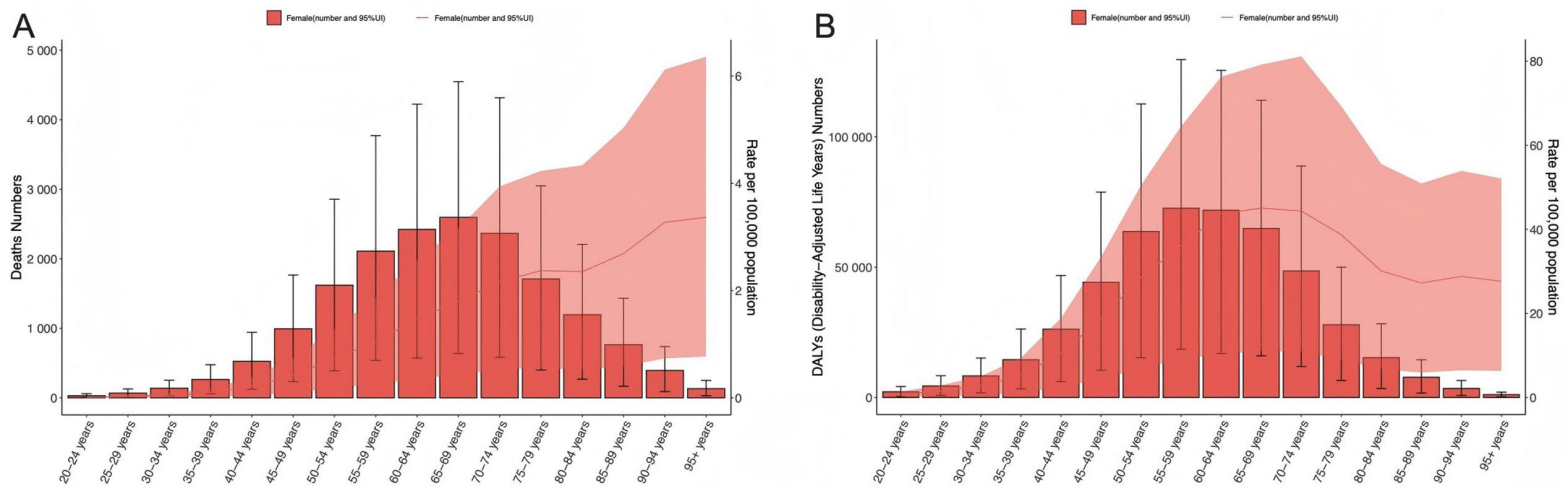
Dual metrics by age group. **(A)** Death counts and ASMR across age groups; **(B)** DALYs and ASDR across age groups. Shaded areas represent 95% uncertainty intervals (95% UI).

Globally in 2021, high BMI-related ovarian cancer deaths peaked in the 65–69 age group, while DALYs peaked in the 55–59 age group. For ASMR and ASDR, global data showed that ASMR increased progressively with age, peaking in the 95+ age group, whereas ASDR peaked in the 65–69 age group (Figure 3).

**Figure 4:**
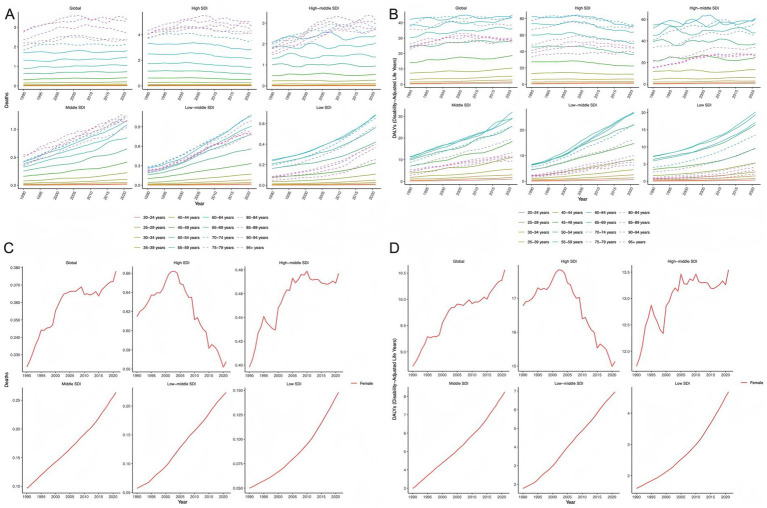
Age-period trends of high BMI-attributable ovarian cancer burden across different age groups. **(A, B)** Temporal trends of mortality rates and DALY rates across age groups in global and five SDI regions; **(C, D)** temporal trends of ASMR and ASDR across age groups in global and five SDI regions.

Age-period analysis showed that mortality and DALYs increased with age across all SDI levels, with the steepest increase seen in adults aged >60 year. From low to high SDI regions, mortality and DALYs exhibited distinct trends: lower SDI regions demonstrated faster growth rates, while high SDI regions showed more gradual changes. Notably, mortality and DALYs in younger populations varied substantially by region, with the most marked increases in low-middle and low SDI regions (Figures 4A, B). In contrast to high SDI regions—where ASMR and ASDR peaked in 2000 and then declined significantly—high-middle, middle, low-middle, and low SDI regions showed overall upward trends consistent with the global pattern. Among these, middle, middle-low and low SDI regions exhibited consistent annual increases in ASMR and ASDR across all age groups with relatively rapid growth rates, while high-middle SDI regions demonstrated a notable decline in ASMR and ASDR around 1995 followed by a gradual upward trend after 2000 (Figures 4C,D).

### 3.5 Association between SDI and high BMI-attributable ovarian cancer burden

**Figure 5:**
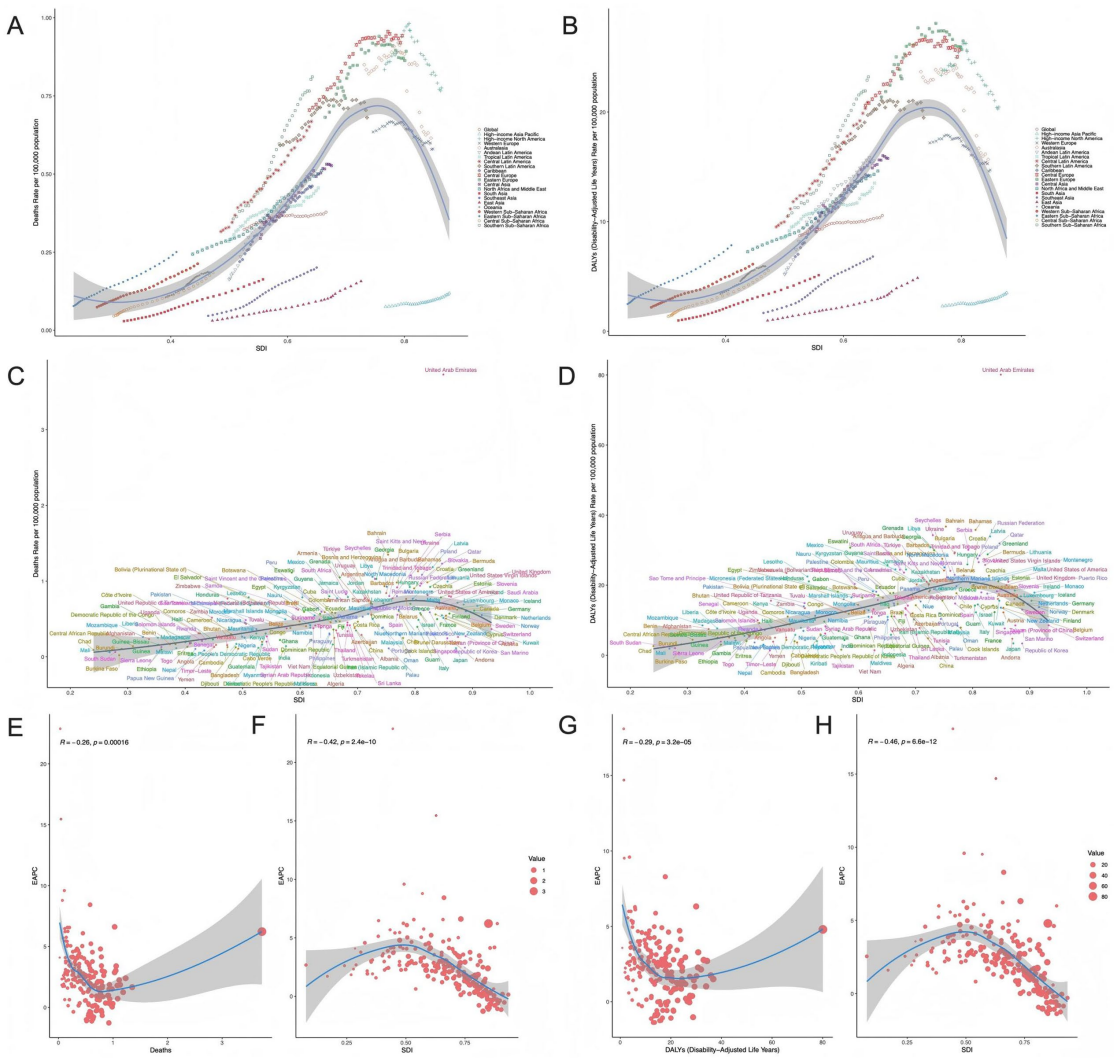
Association between SDI and high BMI-attributable ovarian cancer burden. **(A, B)** Trends of ASMR and ASDR with SDI across 21 regions; **(C, D)** Trends of ASMR and ASDR with SDI among 204 countries; **(E, F)** Trends of EAPC with mortality rates and SDI; **(G, H)** Trends of EAPC with DALY rates and SDI.

Globally and across 21 GBD regions, ASMR and ASDR of ovarian cancer attributable to high BMI exhibited non-linear relationships with SDI, showing an overall declining trend with increasing SDI values. However, in sub-Saharan Africa, despite having low SDI levels, the region maintained relatively high ASMR and ASDR (Figures 5A,B). Among 204 countries, ASMR and ASDR of high BMI-related ovarian cancer decreased as SDI increased. Notably, the United States showed higher-than-expected ASMR given its high SDI level, despite having lower mortality rates compared to most global regions. China maintained relatively low ASMR within the low-to-middle SDI range. Countries such as Russia and Brazil displayed substantial DALY burdens despite their higher SDI levels (Figures 5C,D).

In 2021, EAPC was significantly negatively correlated with ASMR (*R* = −0.26; *p* < 0.001) and ASDR (*R* = −0.29; *p* < 0.001), with regions having higher ASMR/ASDR generally having lower EAPC values. An outlier in the EAPC-ASMR/ASDR relationship represented a region with relatively high mortality but exhibited an unusually large EAPC value, indicating exceptionally rapid mortality decline (Figures 5E,G). Furthermore, negative correlations existed between SDI and EAPC of both ASMR (*R* = −0.42) and ASDR (*R* = −0.46) (*p* < 0.001). Lower SDI regions generally demonstrated higher EAPC values with greater variability, while EAPC became more stable with increasing SDI (Figures 5F,H).

### 3.6 AAPC and APC analysis of high BMI-attributable ovarian cancer burden

**Figure 6:**
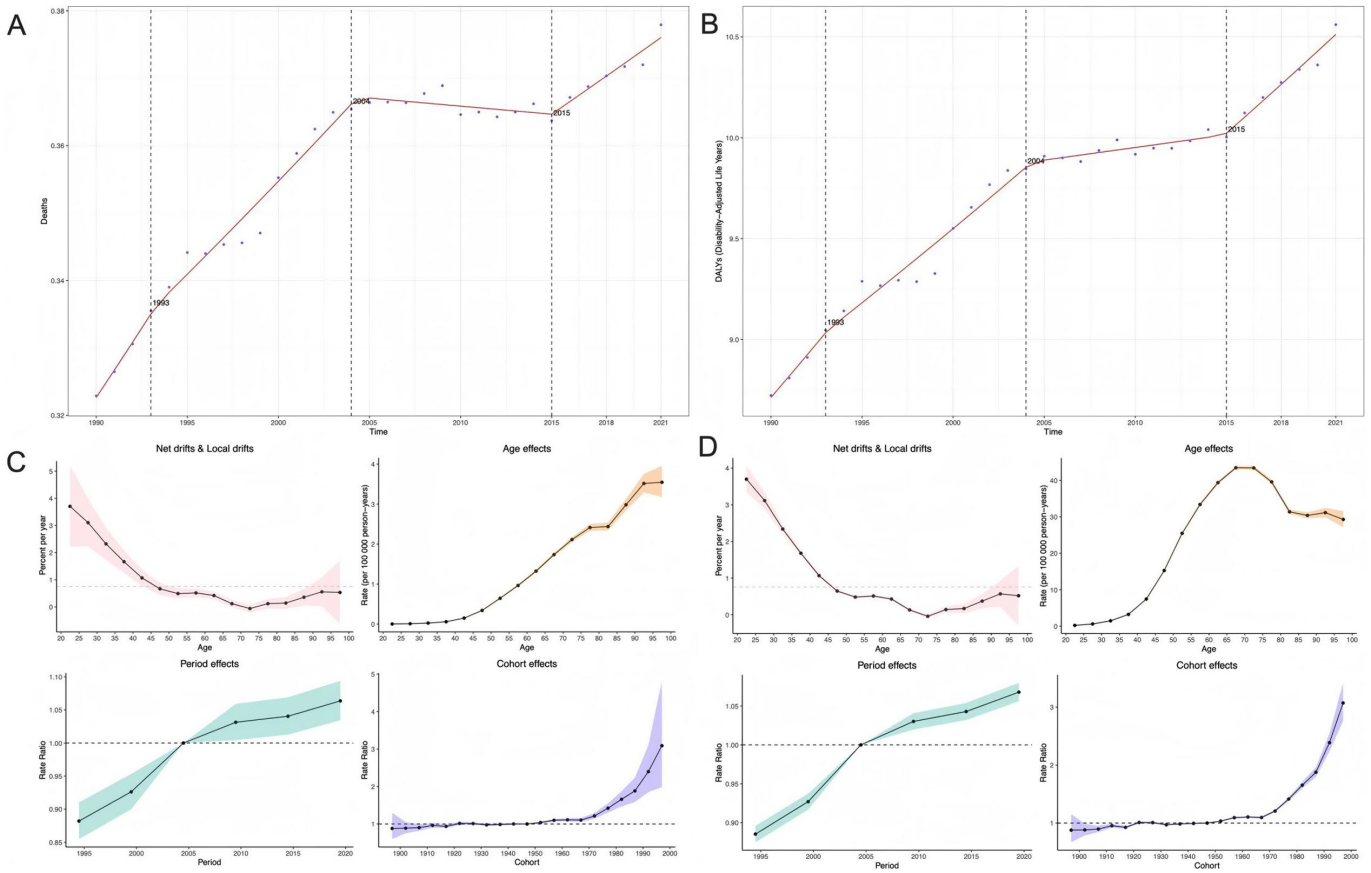
AAPC and APC analysis of high BMI-attributable ovarian cancer burden. **(A, B)** Temporal trends in mortality rates and DALY rates for the overall population; **(C, D)** Age-period-cohort effects on mortality rates and DALY rates. Net drifts and local drifts: Validate dynamic disease risk changes with age; Age effect: Characterizes risk progression with advancing age; Period effect: Assesses temporal influences on disease risk; Cohort effect: Reveals long-term risk impacts across birth generations.

Overall, both ASMR (AAPC = 0.002, 95% CI: 0.002–0.002) and ASDR (AAPC = 0.058, 95% CI: 0.054–0.062) showed significant increasing trends (*p* < 0.05). Three key inflection points were identified in 1993, 2004, and 2015. ASMR increased significantly between 1990 and 2004 (APC = 1.213; 95% CI: 0.698–1.731; *p* < 0.05). The upward trend in ASMR weakened between 2004 and 2015 but was not statistically significant (*p* > 0.05). After 2015, ASMR resumed an upward trend with substantial data variability. For ASDR, the upward trend continued between 2004 and 2015 but at a slower rate. After 2015, DALYs maintained a stable upward trajectory with relatively low data dispersion (Figures 6A, B).

Age-period-cohort analysis showed significant age, period, and cohort (APC) effects on both ASMR and ASDR. The age-specific mortality and DALY rates initially declined sharply before transitioning to gradual increases, with 70 years as the inflection point. ASMR demonstrated a consistent and pronounced age-dependent increase from 25 to 95 years, while ASDR peaked at 20–65 years before declining with advancing age. Period rate ratios (RRs) for both ASMR and ASDR were <1 between 1995 and 2005, then exceeded 1 and continued to increase. For birth cohorts, RRs for ASMR and ASDR exceeded 1 after 1960, with significant upward trends and higher relative risks in later cohorts (Figures 6C, D).

### 3.7 BAPC projection of high BMI-attributable ovarian cancer burden

**Figure 7:**
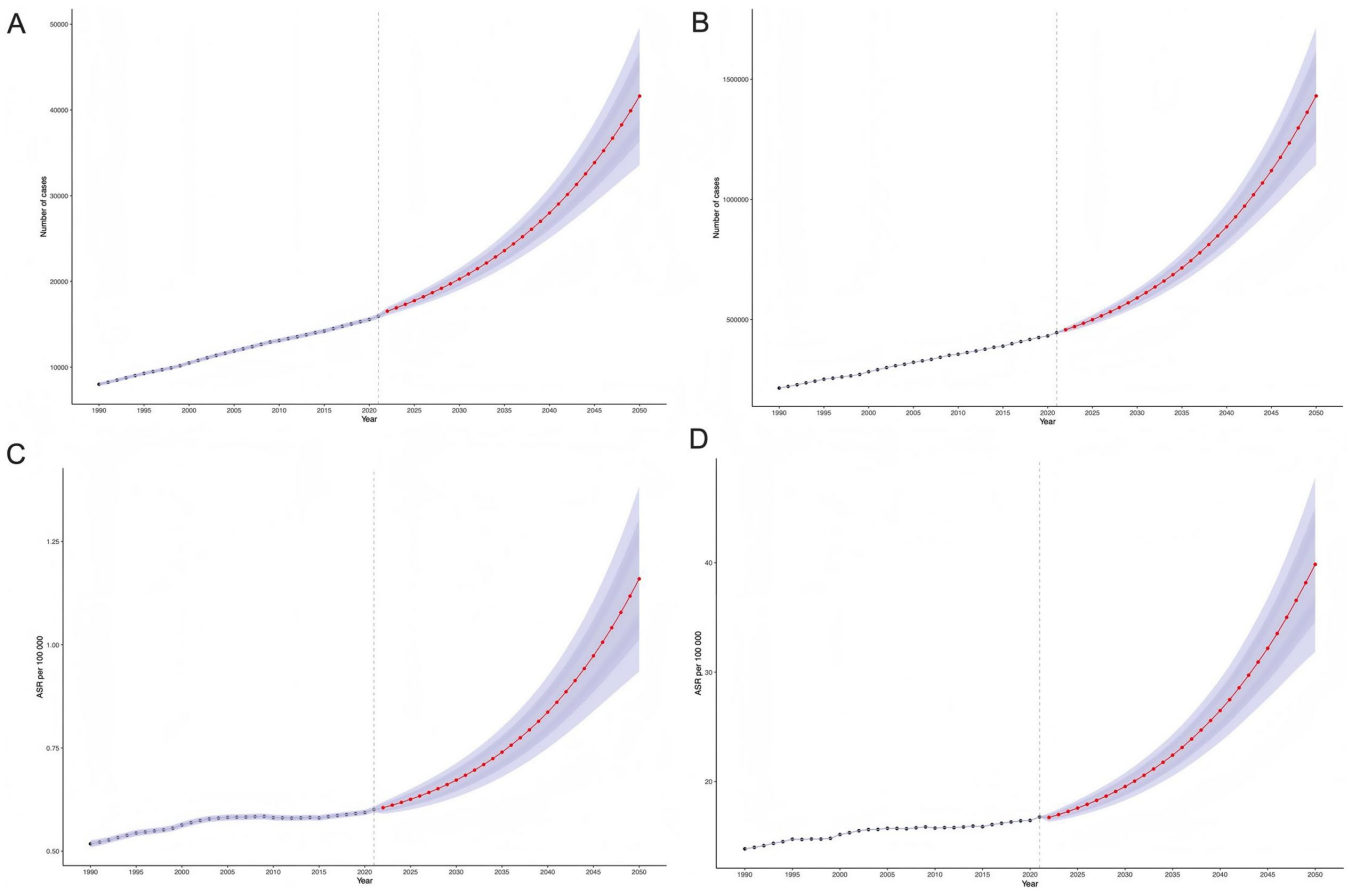
BAPC projections of high BMI-attributable ovarian cancer burden. **(A, B)** Projected death counts and DALY numbers for the general population through 2050; **(C, D)** Projected ASMR and ASDR for the general population through 2050.

During 1990–2020, death counts and DALY numbers showed relatively stable growth, but exhibited accelerated increases after 2020. Projections suggest these will reach 42,000 deaths and 1.4 million DALYs by 2050 (Figures 7A, B). Concurrently, ASMR and ASDR are projected to rise significantly during 2020–2050, reaching 1.2 per 100,000 and 40 per 100,000, respectively, by 2050 (Figures 7C, D).

### 3.8 Decomposition analysis of ovarian cancer burden attributable to high BMI

We performed decomposition analyses of high BMI-related ovarian cancer deaths and DALYs at the global, SDI-stratified, and 21 regional levels. This analysis quantified the numerical contributions of aging, epidemiological transition, and population growth to deaths and DALYs, and identified differences in these factors’ effects across levels.

At the global level from 1990 to 2021, there were approximately 9,500 additional deaths, with population factors contributing 5,500 (57.9%), aging contributing 2,800 (29.5%), and epidemiological changes contributing 1,200 (12.6%). Over the same period, global DALYs increased by 250,000: population growth contributed 120,000 (48.0%), aging contributed 70,000 (28.0%), and epidemiological changes contributed 60,000 (24.0%).

**Figure 8:**
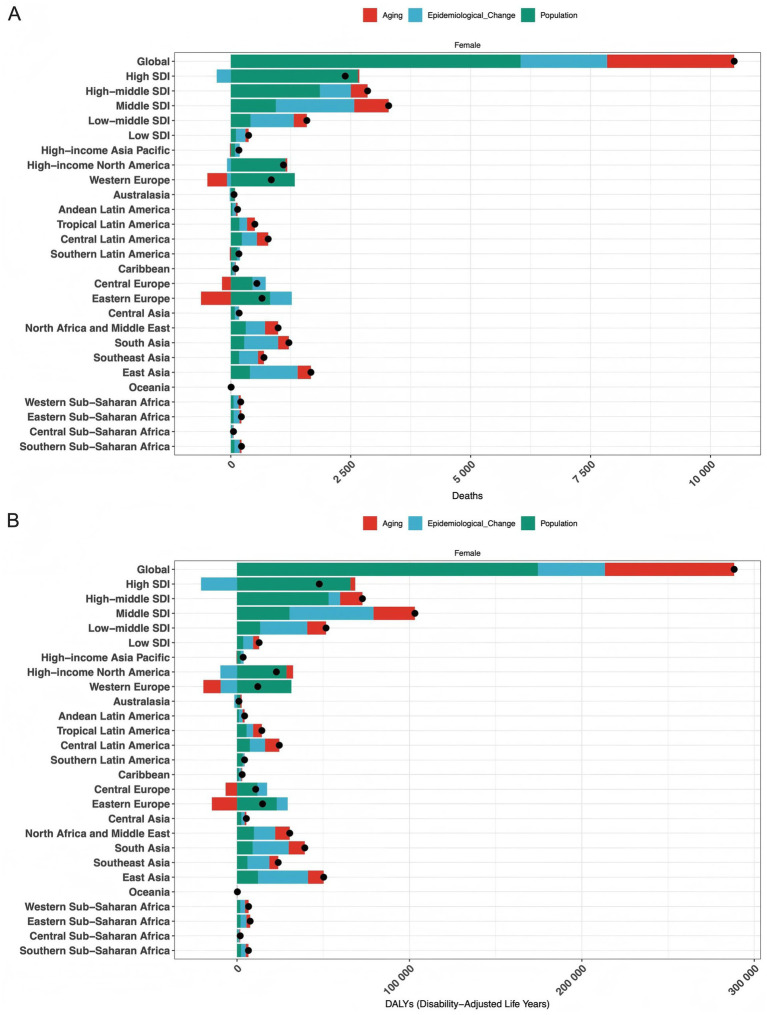
Decomposition analysis of death counts and DALY numbers at global, five SDI-level, and 21 regional levels. **(A)** Decomposition of additional deaths by three factors: aging (red segments), epidemiological change (blue segments), and population growth (green segments); black dots indicate reference data points. **(B)** Decomposition of increased DALYs by the same three factors, with consistent color coding and reference dots.

Among SDI regions, aging had a prominent impact on the health burden in high SDI regions, contributing approximately 53.3% (800/1,500) of deaths and 51.4% (18,000/35,000) of DALYs. In low SDI regions, epidemiological transition contributed about 33.3% of both deaths (200/300) and DALYs (4,000/12,000). Additionally, these factors’ effects varied by region: aging significantly contributed to deaths and DALYs in high-income Asia Pacific and Western Europe; population growth dominated in parts of Latin America and sub-Saharan Africa; and epidemiological transition also had notable impacts (Figure 8).

### 3.9 Health inequality in high BMI-attributable ovarian cancer burden

**Figure 9:**
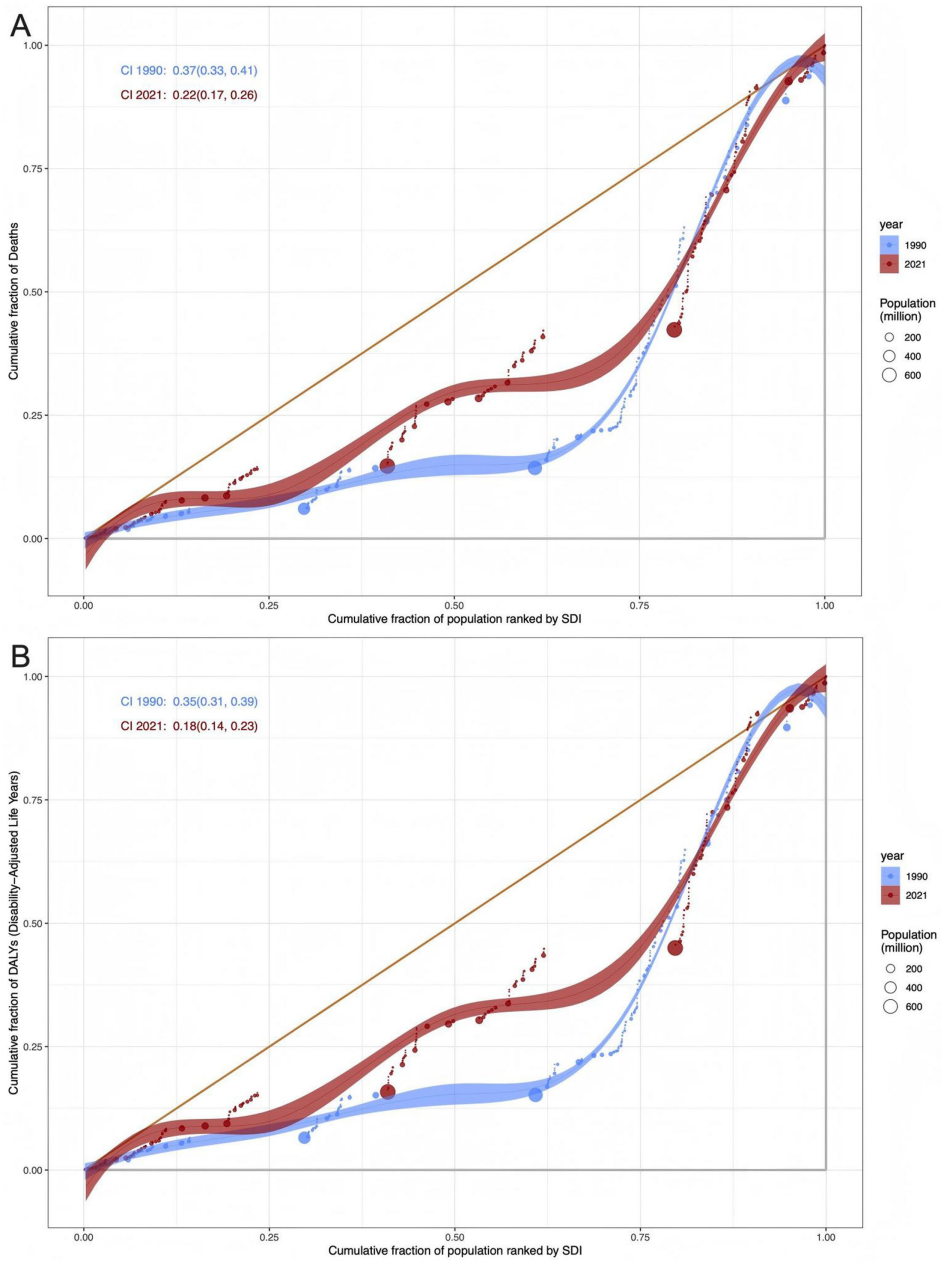
Health inequalities in high BMI-attributable ovarian cancer burden. **(A)** Lorenz curve showing cumulative fraction of deaths versus cumulative fraction of population ranked by SDI (1990: blue line; 2021: red line); shaded areas represent 95% confidence intervals, and dot size reflects population size. **(B)** Lorenz curve for cumulative fraction of DALYs against SDI (1990: blue line; 2021: red line), with the same visual specifications as Panel A.

Data from 1990 to 2021 showed that while ASMR and ASDR remained positively associated with SDI rankings, the gap between SDI-stratified groups narrowed significantly. The concentration index (CI) declined from 0.37 (95% CI: 0.33, 0.41) in 1990 to 0.22 (95% CI: 0.17, 0.26) in 2021, reflecting reduced disparity. Although ASMR and ASDR still increased with SDI, data distribution became more concentrated, indicating narrowing mortality disparities between SDI regions. For example, in regions with low SDI rankings (near 0), ASMR was approximately 0.3/100,000 in 1990 and 0.25/100,000 in 2021; ASDR was approximately 10/100,000 in 1990 and 8/100,000 in 2021. In regions with high SDI rankings (near 1), ASMR was approximately 0.7/100,000 in 1990 and 0.8/100,000 in 2021; ASDR was approximately 20/100,000 in 1990 and 22/100,000 in 2021 (Figure 9).

### 3.10 Frontier analysis of high BMI-attributable ovarian cancer burden

**Figure 10:**
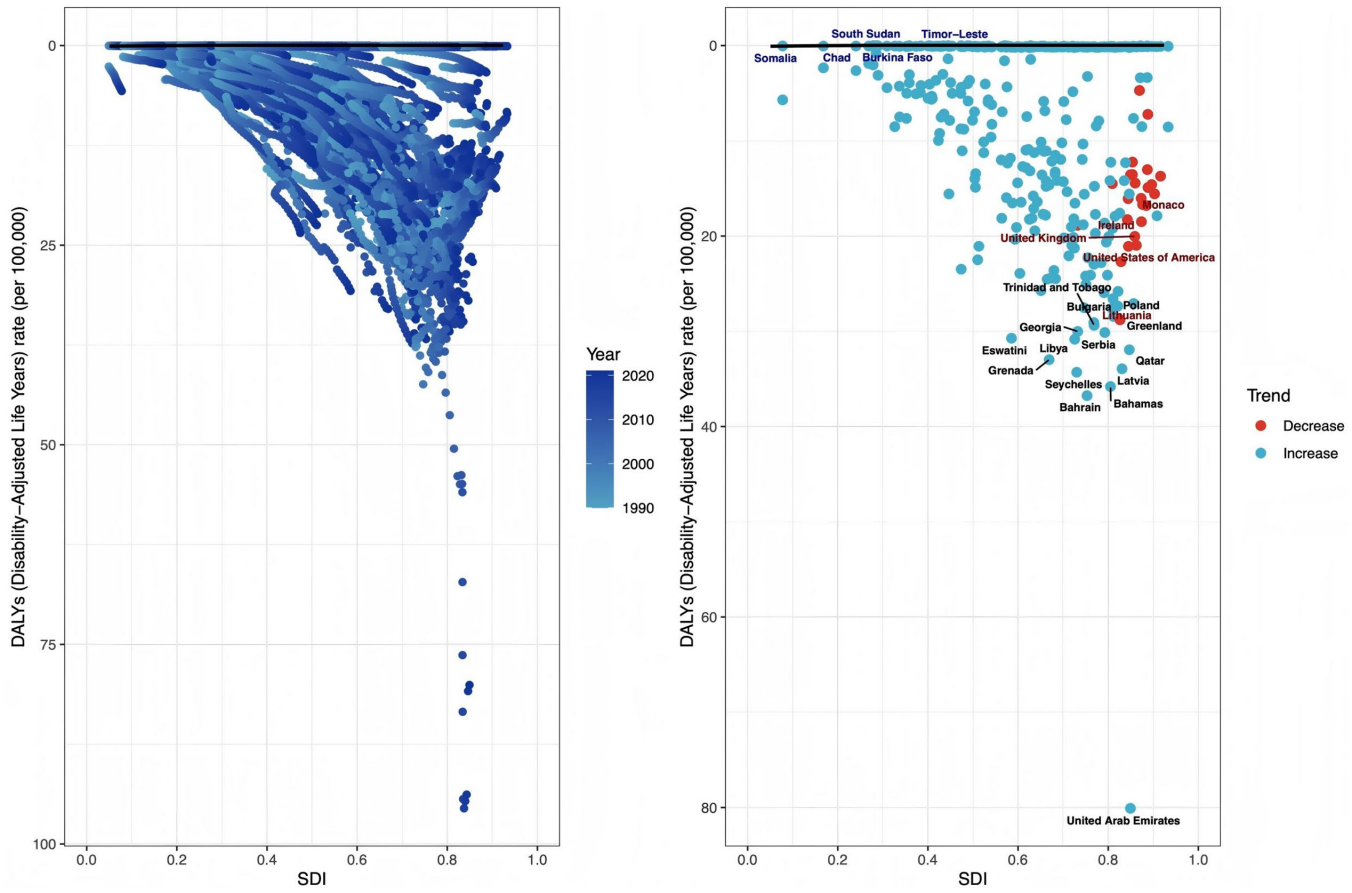
Frontier analysis of high BMI-attributable ovarian cancer burden. (Left panel) Scatter plot where each dot represents a country; the black line denotes the frontier (minimum achievable disease burden), and dot colors (light to dark blue) indicate temporal progression from 1990 to 2021. (Right panel) Subgroup analysis: black-labeled dots: top 15 countries/regions with largest frontier gaps; blue-labeled dots: 5 low-SDI countries (<0.50) with smallest frontier gaps; red-labeled dots: 5 high-SDI countries (>0.85) with largest frontier gaps; red dots: countries with decreasing disease burden; blue dots: countries with increasing disease burden.

By analyzing trends in DALY rates and their gaps relative to the frontier across countries (1990–2021), we assessed regional performance and potential in controlling disease burden. Overall, SDI showed negative correlation with DALY rates, with most countries exhibiting gaps from the frontier. The top 15 countries/regions with largest frontier gaps were: Bahrain, Bahamas, Latvia, Seychelles, Qatar, Grenada, Libya, Eswatini, Serbia, Georgia, Greenland, Bulgaria, Poland, and Trinidad and Tobago. Notably, among high-SDI countries (>0.85), the five with largest frontier gaps were: Lithuania, the United States of America, United Kingdom, Ireland, and Monaco; while among low-SDI countries (<0.50), the five with smallest frontier gaps were: Somalia, Chad, Burkina Faso, South Sudan, and Timor-Leste (Figure 10).

## 4 Discussion

### 4.1 Overall global trends in high BMI-associated ovarian cancer burden

This study found that globally, the number of ovarian cancer deaths caused by high BMI increased from 6,850 to 17,344, and DALYs rose from 188,900 to 477,200 between 1990 and 2021. The estimated annual percentage changes (EAPC) for ASMR and ASDR were 0.40 and 0.51, respectively, indicating a sustained growth in the burden. This trend is consistent with the global epidemiological characteristics of obesity over the same period: data published in The Lancet showed that the global adult obesity rate continued to rise between 1990 and 2021, with a notable acceleration in recent decades, and the adolescent obesity rate increased at a faster pace than the adult population (7).

Growing evidence supports plausible biological mechanisms through which high BMI may contribute to the occurrence and progression of ovarian cancer, though these pathways require further validation in clinical settings. These mechanisms may operate through multiple interconnected pathways. Firstly, hormonal disorders: adipose tissue is an important site for estrogen synthesis. Women with high BMI have elevated estrogen levels (16), which can stimulate the proliferation of ovarian epithelial cells and increase the risk of carcinogenesis (17). Meanwhile, obesity-related insulin resistance can activate the insulin-like growth factor (IGF) pathway, promoting cancer cell invasion and metastasis (18). For example, ovarian cancer cells show increased adhesion to mesothelial explants removed from diet-induced obese model mice, which promotes cancer cell invasion and metastasis (19). In addition, chronic inflammation also plays an important role: obesity-induced hypoxia in adipose tissue can trigger local inflammatory responses, releasing pro-inflammatory factors such as tumor necrosis factor-*α* (TNF-*α*) and interleukin-6 (IL-6) (20), which inhibit the immune system’s surveillance function against tumor cells (21). It is worth noting that metabolomic changes in obese patients (such as accumulation of free fatty acids) can affect the expression of tumor suppressor genes through epigenetic regulation, accelerating tumor progression (22). This aligns with the conceptualization of high BMI as a partially modifiable risk factor for ovarian cancer—one influenced by individual behavioral factors (e.g., diet, physical activity) that can be targeted through intervention, while acknowledging its entanglement with non-modifiable determinants (e.g., genetic predisposition, socioeconomic context, obesogenic environments) that constrain individual agency (23).

The rising high BMI-attributable ovarian cancer burden across age groups—particularly the accelerated growth in adults aged 20–44 years (consistent with our age-period trend analysis)—aligns with the global surge in adolescent obesity and the 15–20-year latency of BMI-related hormonal dysregulation and chronic inflammation. Additionally, low-middle SDI regions (ASMR-EAPC = 4.69) show the steepest burden increase, where rising obesity rates and limited intervention resources amplify pro-inflammatory and metabolic dysregulation, directly driving the observed epidemiological pattern.

Notably, the growth in the burden of high BMI-related ovarian cancer stems not only from population growth and aging (decomposition analysis shows that the two collectively contribute approximately 87.4% to the increase in death cases) but also from a direct association with epidemiological factors (contributing 12.6%). This indicates that even when excluding the impact of changes in population structure, the risk of ovarian cancer onset caused by high BMI itself is still on the rise, highlighting the importance of obesity prevention and control in the primary prevention of ovarian cancer.

### 4.2 Key findings on regional and socioeconomic differences

The regional heterogeneity of high BMI-attributable ovarian cancer burden, supported by our trend and decomposition analyses, provides a crucial basis for formulating targeted strategies with three distinct regional patterns. Western Europe (with the largest number of deaths) and high-income North America (with the highest DALYs) are high-burden regions, where relatively high obesity rates and population aging—identified as key drivers in our data—contribute to their persistent high absolute burden. Despite abundant medical resources, external evidence indicates that the early diagnosis rate of ovarian cancer in these regions remains below 20% and the 5-year survival rate of advanced-stage patients is approximately 50%, which may exacerbate DALY burdens (1, 4). South Asia represents a rapidly growing region, with the largest increases in ASMR and ASDR (EAPCs of 5.86 and 5.61, respectively, from our study data). This trend is closely linked to lifestyle westernization amid recent economic transformation, as the ICMR-INDIAB 2015 study reports prevalence rates of obesity and central obesity ranging from 11.8 to 31.3% and 16.9 to 36.3%, respectively, driven by increased dietary calorie intake and reduced physical activity associated with urbanization (24). Australasia is a declining region, showing downward trends in ASMR and ASDR (EAPCs of −0.96 and −1.25, respectively, from our study data). This pattern is plausibly associated with long-term implementation of obesity prevention and control policies, such as Australia’s ‘healthy food tax’ and New Zealand’s school nutrition intervention programs (25, 26), though our study does not directly evaluate the efficacy of these policies. Results from SDI stratification, based on our study data, show that the burden increase in middle-low and low SDI regions is significantly higher than that in high SDI regions—for example, the ASMR-EAPC of middle-low SDI regions is 4.69 compared with −0.36 in high SDI regions. This difference reflects the dual effects of the ‘health transition’: high SDI regions have alleviated age-standardized rates through public health interventions such as obesity screening and weight loss services, alongside advances in medical technology supported by external evidence (25), but their large population base and aging demographic maintain a high absolute burden. In contrast, middle-low and low SDI regions face ‘two-way growth’ driven by rising obesity rates and limited medical resources. Notably, our data reveal a non-linear negative correlation between SDI and ASMR/ASDR, yet low SDI regions such as sub-Saharan Africa exhibit abnormally high burdens. This observation suggests the presence of synergistic risks beyond economic factors, including infections and nutritional imbalances (27), a hypothesis that requires targeted research.

### 4.3 Age characteristics and predictive trends

This study found that the number of deaths from high BMI-related ovarian cancer peaks at 65–69 years old, DALYs peak at 55–59 years old, and age-standardized rates increase with age (the ASMR is the highest in the 95+ age group). This characteristic is consistent with the natural course of ovarian cancer: the risk of ovarian cancer increases with age, and age, as an independent risk factor, has a synergistic effect with high BMI (6, 28). Hormonal disorders related to obesity and decline in immune function in the elderly may accelerate the progression of ovarian cancer (29, 30). In addition, the peak of DALYs at 55–59 years old suggests that ovarian cancer has a more significant impact on the health of the working-age population, which is related to the social role of women in this age group (such as being the economic pillar of the family). Premature death and disability caused by the disease will increase the family and socioeconomic burden (31).

Predictions from the Bayesian Age-Period-Cohort (BAPC) model show that without intervention measures, the global number of deaths from high BMI-related ovarian cancer will reach 42,000 cases in 2050, and the ASMR will rise to 1.2 per 100,000. This prediction is consistent with the trend predicted by the International Agency for Research on Cancer (IARC) that the number of new ovarian cancer cases worldwide will increase to 445,721 and the number of deaths will increase to 312,617 by 2040 (32). Moreover, special attention should be paid to the phenomenon that the burden on young people (20–44 years old) is increasing rapidly—the rising obesity rate in adolescents (from 1975 to 2016, the number of obese children and adolescents aged 5 to 19 worldwide increased 10-fold, from 11 million to 124 million. It is worth noting that as of 2016, the obesity rate among girls was close to 6%, about 50 million (33)) may lead to an earlier age of onset of ovarian cancer in the future (34).

### 4.4 Health inequality and implications for prevention and control

The analysis of health inequality shows that from 1990 to 2021, the gap in ASMR and ASDR between regions with different SDIs narrowed (the concentration index decreased from 0.37 to 0.22), but the absolute burden in high SDI regions remains high. The frontier analysis identified countries such as Bulgaria and the United States that have a large gap from the ideal control level, as well as countries with good performance in low SDI regions such as Somalia and Chad, providing reference benchmarks for formulating regional strategies. For high SDI countries: although the United States has a high SDI, its ASMR is higher than expected, which may be related to racial differences (the obesity rate and ovarian cancer mortality rate of African-American women are higher than those of white women) and unequal distribution of health resources. It is necessary to strengthen obesity intervention and cancer screening for vulnerable groups. For low SDI countries: the low burden in countries such as Somalia may be related to the low obesity rate (<5%), but it is necessary to be alert to the potential risk of rising obesity rates after economic development, and it is recommended to implement primary prevention in advance (such as school nutrition education).

Based on the results of this study, targeted prevention and control suggestions are put forward. Primary prevention: For middle-low SDI regions, promote community-based ‘sugar reduction and exercise increase’ interventions with local adaptability, such as South Africa’s ‘Walking for Health’ program—this initiative organizes community-led group walking activities 3 times a week, combined with on-site nutrition guidance (e.g., recommending local low-cost whole grains and vegetables), and has been proven to reduce the overweight rate of participating women by 12% within 2 years (35). For low SDI regions, garden-based nutrition education (e.g., the ‘LA Sprouts’ intervention) is a feasible primary prevention model. This 12-week program combined gardening, nutrition, and cooking classes, boosting adolescents’ vegetable preference (16% more in overweight/obese subgroups). Its low-cost, community-engaged design leverages local land and accessible crops, making it highly adaptable to resource-limited low SDI regions for long-term obesity and ovarian cancer prevention (36). For high SDI regions, strengthen ovarian cancer risk screening for women with obesity combined with polycystic ovary syndrome (PCOS), such as setting up specialized clinics in community health centers to conduct annual BMI monitoring and CA125 detection. Secondary prevention: Optimize ovarian cancer screening strategies for high BMI populations (such as CA125 combined with ultrasound examination), referring to the UK’s ‘annual screening for high-risk groups’ model (37); in addition, optimize the treatment level: promote minimally invasive technologies such as laparoscopic surgery in resource-limited regions to reduce treatment-related DALYs, and explore the survival benefits of ‘weight loss combined with cancer treatment’ for obese ovarian cancer patients (38).

### 4.5 Research limitations

This study has certain limitations: first, GBD data rely on model estimation, and the quality of cancer registration data in some low SDI regions is low, which may affect the accuracy of the results; second, the analysis does not include the interaction between genetic factors (such as BRCA mutations) and high BMI, while previous studies have shown that BRCA mutation carriers have a higher risk of obesity-related ovarian cancer; in addition, the prediction model does not consider the potential impact of future intervention measures, and the results are speculations under the ‘no intervention’ scenario.

## 5 Conclusion

The global burden of ovarian cancer attributable to high BMI continues to grow, with significant disparities across regions, age groups, and socioeconomic contexts. Population growth, aging, and rising obesity rates are key driving factors. Moving forward, targeted prevention and control strategies are needed to reduce health inequalities, with particular attention to obesity interventions in middle-low SDI regions and young populations, to curb the further spread of disease burden. This study provides important quantitative evidence for global ovarian cancer prevention and public health decision-making.

## Data Availability

The original contributions presented in the study are included in the article/Supplementary material, further inquiries can be directed to the corresponding author.

## References

[1] Bray F Laversanne M Sung H Ferlay J Siegel RL Soerjomataram I . Global cancer statistics 2022: GLOBOCAN estimates of incidence and mortality worldwide for 36 cancers in 185 countries. CA Cancer J Clin. (2024) 74:229–63. doi: 10.3322/caac.21834, 38572751

[2] Bast RC Jr Lu Z Han CY Lu KH Anderson KS Drescher CW . Biomarkers and strategies for early detection of ovarian cancer. Cancer Epidemiol Biomarkers Prev. (2020) 29:2504–12. doi: 10.1158/1055-9965.EPI-20-1057, 33051337 PMC7710577

[3] Webb PM Jordan SJ. Global epidemiology of epithelial ovarian cancer. Nat Rev Clin Oncol. (2024) 21:389–400. doi: 10.1038/s41571-024-00881-3, 38548868

[4] Parekh N Chandran U Bandera EV. Obesity in cancer survival. Annu Rev Nutr. (2012) 32:311–42. doi: 10.1146/annurev-nutr-071811-150713, 22540252 PMC3807693

[5] Partridge EE Barnes MN. Epithelial ovarian cancer: prevention, diagnosis, and treatment. CA Cancer J Clin. (1999) 49:297–320. doi: 10.3322/canjclin.49.5.297, 11198956

[6] Gaitskell K Hermon C Barnes I Pirie K Floud S Green J . Ovarian cancer survival by stage, histotype, and pre-diagnostic lifestyle factors, in the prospective UK million women study. Cancer Epidemiol. (2022) 76:102074. doi: 10.1016/j.canep.2021.102074, 34942490 PMC8785125

[7] GBD 2021 Adolescent BMI Collaborators. Global, regional, and national prevalence of child and adolescent overweight and obesity, 1990-2021, with forecasts to 2050: a forecasting study for the global burden of disease study 2021. Lancet. (2025) 405:785–812. doi: 10.1016/S0140-6736(25)00397-6 40049185 PMC11920006

[8] Moufarrij S. O’Cearbhaill RE. Novel Therapeutics in Ovarian Cancer: Expanding the Toolbox. Curr Oncol. (2023) 31:97–114. doi: 10.3390/curroncol31010007 38248092 PMC10814452

[9] Rubino F Cummings DE Eckel RH Cohen RV Wilding JPH Brown WA . Definition and diagnostic criteria of clinical obesity. Lancet Diabetes Endocrinol. (2025) 13:221–62. doi: 10.1016/S2213-8587(24)00316-4, 39824205 PMC11870235

[10] Kalliala I Markozannes G Gunter MJ . Obesity and gynaecological and obstetric conditions: umbrella review of the literature. BMJ. (2017) 359:j4511. doi: 10.1136/bmj.j4511 29074629 PMC5656976

[11] Calle EE Rodriguez C Walker-Thurmond K Thun MJ. Overweight, obesity, and mortality from cancer in a prospectively studied cohort of US adults. N Engl J Med. (2003) 348:1625–38. doi: 10.1056/nejmoa021423, 12711737

[12] Zhang Y Daquinag AC Amaya-Manzanares F Sirin O Tseng C Kolonin MG. Stromal progenitor cells from endogenous adipose tissue contribute to pericytes and adipocytes that populate the tumor microenvironment. Cancer Res. (2012) 72:5198–208. doi: 10.1158/0008-5472.CAN-12-0294, 23071132

[13] Zheng Q Banaszak L Fracci S Basali D Dunlap SM Hursting SD . Leptin receptor maintains cancer stem-like properties in triple negative breast cancer cells. Endocr Relat Cancer. (2013) 20:797–808. doi: 10.1530/ERC-13-0329, 24025407 PMC3843956

[14] Park EJ Lee JH Yu GY He G Ali SR Holzer RG . Dietary and genetic obesity promote liver inflammation and tumorigenesis by enhancing IL-6 and TNF expression. Cell. (2010) 140:197–208. doi: 10.1016/j.cell.2009.12.052, 20141834 PMC2836922

[15] Desharnais L Walsh LA Quail DF. Exploiting the obesity-associated immune microenvironment for cancer therapeutics. Pharmacol Ther. (2022) 229:107923. doi: 10.1016/j.pharmthera.2021.107923, 34171329

[16] Leeners B Geary N Tobler PN Asarian L. Ovarian hormones and obesity. Hum Reprod Update. (2017) 23:300–21. doi: 10.1093/humupd/dmw045, 28333235 PMC5850121

[17] Wang Y Chang H Li X Zhang H Zhou Q Tang S . Estrogen regulates PDPK1 to promote cell proliferation in epithelial ovarian cancer. Heliyon. (2024) 10:e40296. doi: 10.1016/j.heliyon.2024.e40296, 39624293 PMC11609661

[18] Park J Morley TS Kim M Clegg DJ Scherer PE. Obesity and cancer--mechanisms underlying tumour progression and recurrence. Nat Rev Endocrinol. (2014) 10:455–65. doi: 10.1038/nrendo.2014.94, 24935119 PMC4374431

[19] Liu Y Metzinger MN Lewellen KA Cripps SN Carey KD Harper EI . Obesity contributes to ovarian cancer metastatic success through increased lipogenesis, enhanced vascularity, and decreased infiltration of M1 macrophages. Cancer Res. (2015) 75:5046–57. doi: 10.1158/0008-5472.CAN-15-0706, 26573796 PMC4668203

[20] Schleh MW Caslin HL Garcia JN . Metaflammation in obesity and its therapeutic targeting. Sci Transl Med. (2023) 15:eadf9382. doi: 10.1126/scitranslmed.adf9382 37992150 PMC10847980

[21] Denk D Greten FR. Inflammation: the incubator of the tumor microenvironment. Trends Cancer. (2022) 8:901–14. doi: 10.1016/j.trecan.2022.07.002, 35907753

[22] Tang C Castillon VJ Waters M Fong C Park T Boscenco S . Obesity-dependent selection of driver mutations in cancer. Nat Genet. (2024) 56:2318–21. doi: 10.1038/s41588-024-01969-3, 39468367 PMC11549034

[23] Sobal J Bisogni CA. Constructing food choice decisions. Ann Behav Med. (2009) 38:S37–46. doi: 10.1007/s12160-009-9124-5 19787306

[24] Ahirwar R Mondal PR. Prevalence of obesity in India: a systematic review. Diabetes Metab Syndr. (2019) 13:318–21. doi: 10.1016/j.dsx.2018.08.032, 30641719

[25] Lee A Patay D Herron LM Parnell Harrison E Lewis M. Affordability of current, and healthy, more equitable, sustainable diets by area of socioeconomic disadvantage and remoteness in Queensland: insights into food choice. Int J Equity Health. (2021) 20:153. doi: 10.1186/s12939-021-01481-8, 34193163 PMC8243618

[26] McKelvie-Sebileau P Swinburn B Glassey R Tipene-Leach D Gerritsen S. Health, wellbeing and nutritional impacts after 2 years of free school meals in New Zealand. Health Promot Int. (2023) 38:daad093. doi: 10.1093/heapro/daad093, 37590384 PMC10434982

[27] Mocumbi AO. Focus on non-communicable diseases: an important agenda for the African continent. Cardiovasc Diagn Ther. (2013) 3:193–5. doi: 10.3978/j.issn.2223-3652.2013.12.07, 24400202 PMC3878115

[28] Engeland A Tretli S Bjørge T. Height, body mass index, and ovarian cancer: a follow-up of 1.1 million Norwegian women. J Natl Cancer Inst. (2003) 95:1244–8. doi: 10.1093/jnci/djg010, 12928351

[29] Priyanka HP Nair RS. Neuroimmunomodulation by estrogen in health and disease. AIMS Neurosci. (2020) 7:401–17. doi: 10.3934/Neuroscience.2020025, 33263078 PMC7701372

[30] Ullah A Chen Y Singla RK Cao D Shen B. Pro-inflammatory cytokines and CXC chemokines as game-changer in age-associated prostate cancer and ovarian cancer: insights from preclinical and clinical studies' outcomes. Pharmacol Res. (2024) 204:107213. doi: 10.1016/j.phrs.2024.107213, 38750677

[31] Hutchinson B Euripides M Reid F Allman G Morrell L Spencer G . Socioeconomic burden of ovarian cancer in 11 countries. JCO Glob Oncol. (2025) 11:e2400313. doi: 10.1200/GO-24-00313, 39977710

[32] World Ovarian Cancer Coalition. World ovarian Cancer coalition data briefing projects loss of over 4 million women to ovarian Cancer by 2040 World Ovarian Cancer Coalition (2023).

[33] NCD Risk Factor Collaboration (NCD-RisC). Worldwide trends in body-mass index, underweight, overweight, and obesity from 1975 to 2016: a pooled analysis of 2416 population-based measurement studies in 128.9 million children, adolescents, and adults. Lancet. (2017), 390:2627–2642. doi: 10.1016/S0140-6736(17)32129-3 29029897 PMC5735219

[34] Recalde M Pistillo A Davila-Batista V Leitzmann M Romieu I Viallon V . Longitudinal body mass index and cancer risk: a cohort study of 2.6 million Catalan adults. Nat Commun. (2023) 14:3816. doi: 10.1038/s41467-023-39282-y, 37391446 PMC10313757

[35] Gradidge PJ Golele PN. Walking as a feasible means of effecting positive changes in BMI, waist, and blood pressure in black south African women. Afr Health Sci. (2018) 18:917–21. doi: 10.4314/ahs.v18i4.10, 30766555 PMC6354885

[36] Gatto NM Martinez LC Spruijt-Metz D Davis JN. LA sprouts randomized controlled nutrition, cooking and gardening programme reduces obesity and metabolic risk in Hispanic/Latino youth. Pediatr Obes. (2017) 12:28–37. doi: 10.1111/ijpo.12102, 26909882 PMC5362120

[37] Rosenthal AN Fraser L Manchanda R Badman P Philpott S Mozersky J . Results of annual screening in phase I of the United Kingdom familial ovarian cancer screening study highlight the need for strict adherence to screening schedule. J Clin Oncol. (2013) 31:49–57. doi: 10.1200/JCO.2011.39.7638, 23213100 PMC3530690

[38] Lim PW Stucky CH Wasif N Etzioni DA Harold KL Madura JA 2nd . Bariatric surgery and longitudinal Cancer risk: a review. JAMA Surg. (2024) 159:331–8. doi: 10.1001/jamasurg.2023.5809, 38294801

