# Supplementary material for: Global, regional, and national burden of ovarian cancer due to high BMI, 1990–2021 and projections to 2050: a systematic analysis based on the global burden of disease 2021 study
Source: Front Nutr. 2026 Feb 9;13:1688767. doi: 10.3389/fnut.2026.1688767 (PMC12926127; doi:10.3389/fnut.2026.1688767)
Supplement: Supplementary file 1 [file Image_1.pdf]

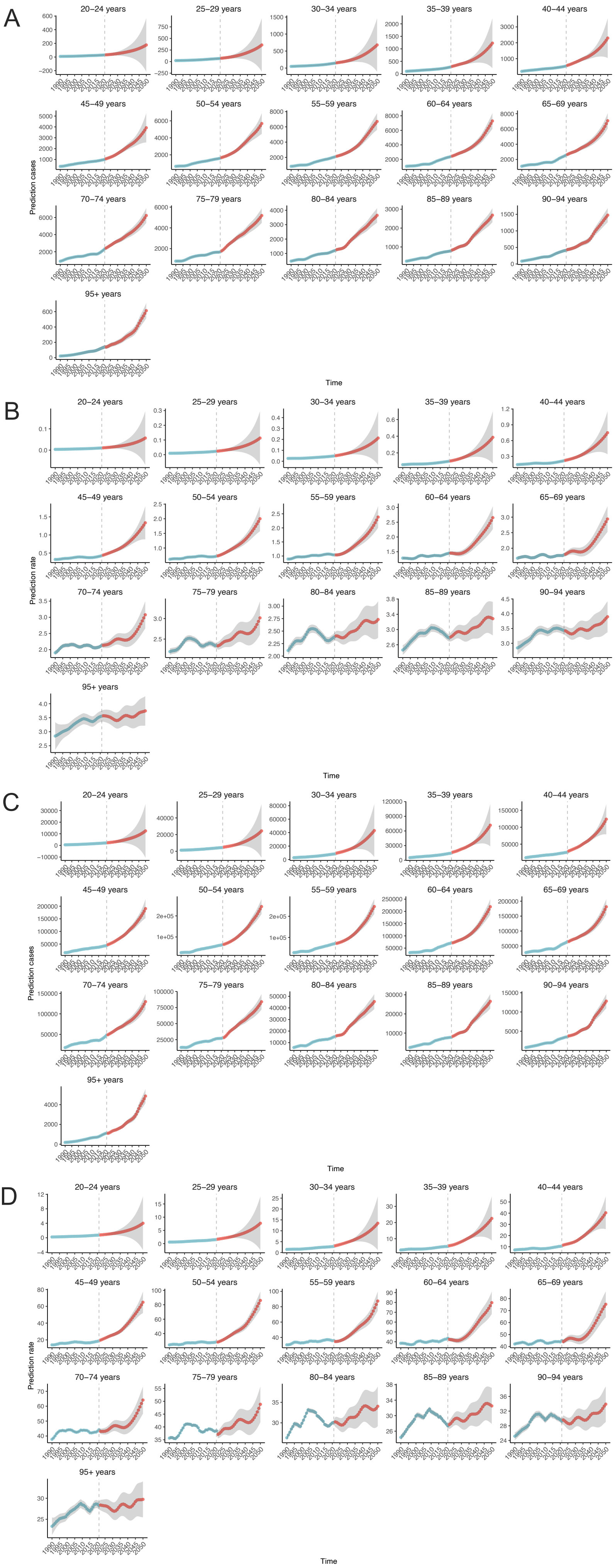

Figure 1 attached. Projections of global high BMI-related ovarian cancer burden up to 2050 based on the Bayesian Age-Period-Cohort (BAPC) model:
A. Mortality rates by age group; B. ASMR by age group; C. DALYs rates by age group; D. ASDR by age group.
